# Aberrant Gene Expression in Humans

**DOI:** 10.1371/journal.pgen.1004942

**Published:** 2015-01-24

**Authors:** Yong Zeng, Gang Wang, Ence Yang, Guoli Ji, Candice L. Brinkmeyer-Langford, James J. Cai

**Affiliations:** 1 Department of Automation, Xiamen University, Xiamen, Fujian, China; 2 Department of Veterinary Integrative Biosciences, Texas A&M University, College Station, Texas, United States of America; 3 Innovation Center for Cell Biology, Xiamen University, Xiamen, Fujian, China; 4 Interdisciplinary Program in Genetics, Texas A&M University, College Station, Texas, United States of America; Stanford University, UNITED STATES

## Abstract

Gene expression as an intermediate molecular phenotype has been a focus of research interest. In particular, studies of expression quantitative trait loci (eQTL) have offered promise for understanding gene regulation through the discovery of genetic variants that explain variation in gene expression levels. Existing eQTL methods are designed for assessing the effects of common variants, but not rare variants. Here, we address the problem by establishing a novel analytical framework for evaluating the effects of rare or private variants on gene expression. Our method starts from the identification of outlier individuals that show markedly different gene expression from the majority of a population, and then reveals the contributions of private SNPs to the aberrant gene expression in these outliers. Using population-scale mRNA sequencing data, we identify outlier individuals using a multivariate approach. We find that outlier individuals are more readily detected with respect to gene sets that include genes involved in cellular regulation and signal transduction, and less likely to be detected with respect to the gene sets with genes involved in metabolic pathways and other fundamental molecular functions. Analysis of polymorphic data suggests that private SNPs of outlier individuals are enriched in the enhancer and promoter regions of corresponding aberrantly-expressed genes, suggesting a specific regulatory role of private SNPs, while the commonly-occurring regulatory genetic variants (i.e., eQTL SNPs) show little evidence of involvement. Additional data suggest that non-genetic factors may also underlie aberrant gene expression. Taken together, our findings advance a novel viewpoint relevant to situations wherein common eQTLs fail to predict gene expression when heritable, rare inter-individual variation exists. The analytical framework we describe, taking into consideration the reality of differential phenotypic robustness, may be valuable for investigating complex traits and conditions.

## Author Summary

The uniqueness of individuals is due to differences in the combination of genetic, epigenetic and environmental determinants. Understanding the genetic basis of phenotypic variation is a key objective in genetics. Gene expression has been considered as an intermediate phenotype, and the association between gene expression and commonly-occurring genetic variants in the general population has been convincingly established. However, there are few methods to assess the impact of rare genetic variants, such as private SNPs, on gene expression. Here we describe a systematic approach, based on the theory of multivariate outlier detection, to identify individuals that show unusual or aberrant gene expression, relative the rest of the study cohort. Through characterizing detected outliers and corresponding gene sets, we are able to identify which gene sets tend to be aberrantly expressed and which individuals show deviant gene expression within a population. One of our major findings is that private SNPs may contribute to aberrant expression in outlier individuals. These private SNPs are more frequently located in the enhancer and promoter regions of genes that are aberrantly expressed, suggesting a possible regulatory function of these SNPs. Overall, our results provide new insight into the determinants of inter-individual variation, which have not been evaluated by large population-level cohort studies.

## Introduction

The advent of high-throughput genotyping and sequencing technologies enables a comprehensive characterization of the genomic and transcriptomic landscapes of each individual. Deciphering the massive data points associated with individuals presents a major challenge [1, 2]. Over the last couple of years, eQTL analyses have provided in-depth insights into the effect of genetic variation on regulating gene expression [3–6]. More recently, research has also focused on the contribution of genetic variation on the variance of gene expression [7–9].

The analytical frameworks adopted by most eQTL studies have historically been based on population-level test statistics, which are powerful for establishing associations between commonly-occurring genetic variations and gene expression. However, few frameworks or statistics are available for assessing the impacts of rare genetic variants to gene expression (except, for example, [10]). The problem is further exacerbated by the fact that individual gene expression is a function of both genetic and non-genetic (such as epigenetic and environmental) factors, as well as their combined action. Our failure to detect the effects of rare variants with large effects in biological samples, along with the inherent difficulty in dissecting the complex factors influencing gene expression will hinder efforts to define and prioritize relevant variants and impede the development of improved personalized diagnostic and therapeutic options.

Here, we envision an alternative approach based on the theory of multivariate outliers to address these technical challenges. More specifically, we measure how any two individuals differ in their expression profiles and quantify these differences with respect to a set of genes between individuals. Based on the expression differences, we detect outlier individuals whose expression profiles are so divergent from those of others in the population that the divergence cannot be explained by random sampling variation alone. Many methods of outlier detection have been developed. The most commonly used of these methods, such as those based on the estimation of the location and scatter of the data points or the quantiles of the data, are more applicable to univariate than multivariate settings. In practice, however, phenotypic traits are associated with changes of multiple genes in biological pathways and molecular networks, more often than single gene alterations. Reliably identifying outliers in such a multivariate setting is a challenging problem—unlike the simpler case of univariate outlier detection, simple graphical diagnostic tools like the boxplot often lack statistical power when the analysis of more than one dimension is attempted [11].

To this end, we adapted the multivariate outlier method that allows simultaneous evaluation of expression data with respect to many dimensions derived from multiple genes. With this method, even though there is no natural ordering of multivariate data on which “extremeness” of an observation can be ascertained, outliers showing markedly different data profile can be detected. Using a framework based on this approach, we specifically address the following research questions: Are there any differences between the functional properties of genes tending to (or tending not to) be aberrantly expressed? Is aberrant expression population-specific? What are the roles of genetic and non-genetic factors in aberrant expression? Do common or rare genetic variants contribute to aberrant expression? Our overall results clearly demonstrate that outliers, while often considered as error or noise, do carry important biologically-relevant information. Thus, the careful characterization of the genetic bases underlying the markedly different expression profiles of outlier samples is both worthwhile and necessary. Accurate description of inter-individual expression differences requires the incorporation of the effects of both common and rare regulatory genetic variants.

## Results

### Study overview

The main results of our study comprise three parts. The first part concerns the identification of sets of functionally related genes whose expression discrepancies among individuals are significantly greater (or smaller) than those of random gene sets. The second part concerns the identification of outlier individuals whose expression profiles with respect to gene sets are significantly divergent from those of others in the population. The third part concerns the uncovered evidence that private SNPs contribute to aberrant expression in outlier individuals.

**Figure 1:**
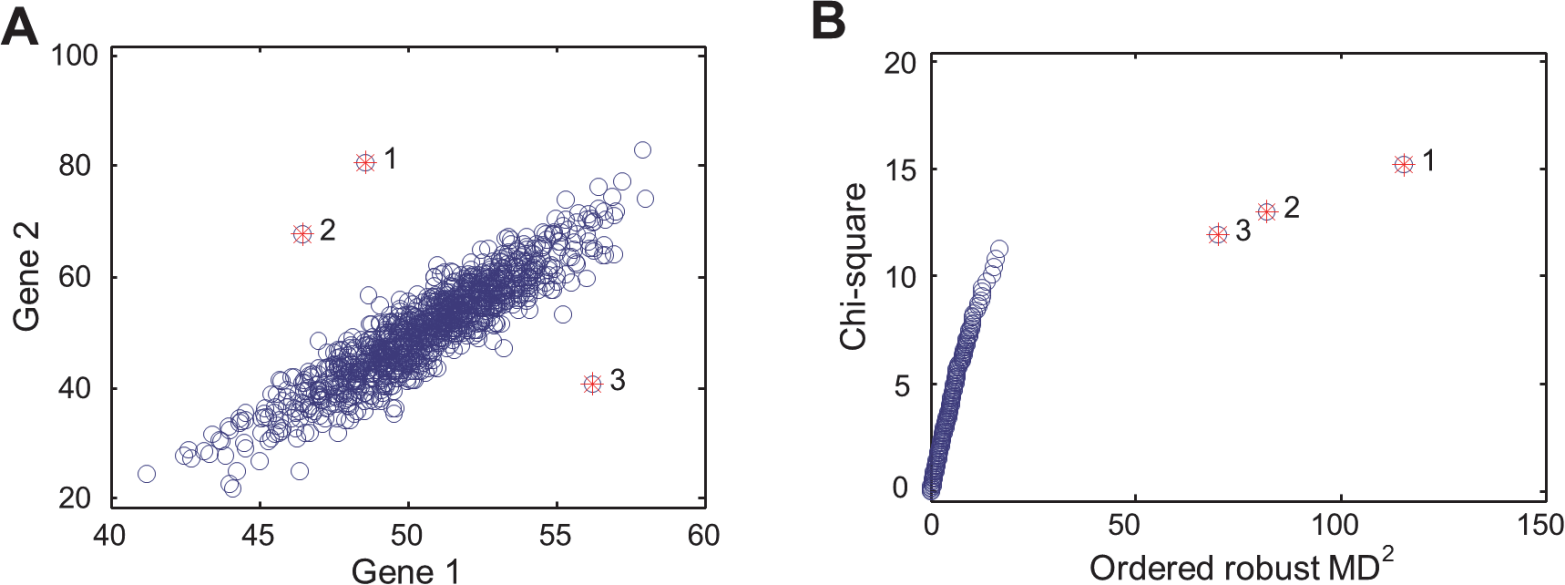
MD-based multivariate outlier detection. (**A**) Scatter plot for the expression levels of two hypothetical genes. Three outliers indicated with red stars have the largest MD values to the population mean. (**B**) The chi-square plot showing the relative position and order of the three outlier data points, compared to those of non-outlier data points.

Data analysis in the first two parts relied on a metric of statistical distance that can quantify the dissimilarities between individuals in the expression levels of gene sets, rather than single gene. To this purpose, we adapted *Mahalanobis distance* (MD), a multivariate metric that can be used to measure the dissimilarity between two vectors [12]. Key features of MD are illustrated in Fig. 1, which shows a hypothetical example of MD, compared to the simple Euclidean distance. Here, the expression levels of two genes are correlated and the Euclidean distance is not an appropriate measure of distance between data points (or individuals). MD, on the other hand, accounts for the correlation through estimating the covariance matrix from the observations, making MD a more appropriate distance statistic. With a given gene set (e.g., the two genes of the hypothetical example), we can calculate MD_i_ for *N* individuals under consideration (*i* = 1 to *N*). Each MD_i_ is the multivariate distance from the individual *i* to the population mean, with the correlation between expression profiles of individuals captured by the inter-individual expression covariance. In Fig. 1A, the top three data points with largest MD_i_ are labeled with 1, 2, and 3, while the Euclidean distances from these data points to the population mean are not the largest. With MD_i_ of each individual, we can calculate the *sum of squared MD_i_* (SSMD). SSMD summarizes the overall distribution of MD_i_ across individuals for the gene set. The squaring operation puts more weight on larger MD_i_ values of outlier individuals. Gene sets with larger SSMD are more likely to contain genes that are aberrantly expressed by outlier individuals. Thus, comparing SSMD values of gene sets, we can identify sets of genes that tend to (or tend not to be) aberrantly expressed (i.e., Part 1 of the main results).

The outlier individuals can be identified with ordered MD_i_. To do so, we used the tool for multivariate outlier recognition, chi-square plot [13]. As seen in Fig. 1B, the three data points with the largest MD_i_ are recognized as outliers. These data points, as shown in Fig. 1A, are the most remote observations with the largest MD_i_ to the population mean. None of the three data points would otherwise be identified as outliers by using Euclidean distance. More important, none of them would otherwise be identified as outliers if we used any univariate approach. This is because that, when the two genes are considered separately, the expression levels of either gene in the three individuals are in the “normal” range. Finally the purpose of identifying outlier individuals is to study the genetic basis of aberrant expression of genes in outliers. That is to say, once the outlier individuals are identified, the genetic variation associated with outlier individuals can be further analyzed to see what kinds of genetic variation contribute to aberrant expression (i.e., Part 2 of the main results).

### Gene sets (L-SSMD) that tend to be aberrantly expressed

We started by identifying gene sets that are more likely to be aberrantly expressed. We obtained the expression data matrix of 10,231 protein-coding genes in 326 lymphoblastoid cell lines (LCLs) of European descent (EUR) from the Geuvadis project RNA-seq study [3]. We used SSMD to measure the total deviation of expression profiles from all individuals to the population mean for gene sets. We computed SSMD for all gene sets with fewer than 150 expressed genes in the Molecular Signatures Database (MSigDB) [14] and the GWAS catalog [15].

**Figure 2:**
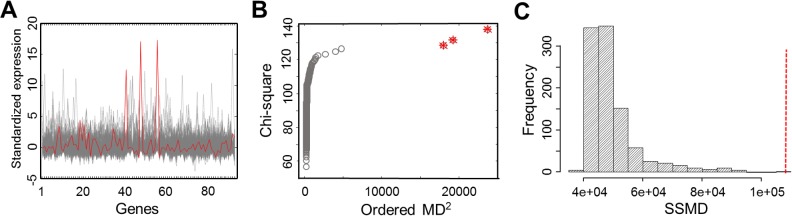
Gene expression profiles and outlier detection in the gene set, G-protein coupled receptor activity. (**A**) The expression profiles of 326 EUR samples for 94 genes in the gene set. The expression profile of the outlier individual with the largest SSMD is outlined in red. (**B**) The chi-square plot showing three outliers, as highlighted with the star symbol. (**C**) The null distribution of SSMD established from 1,000 permutations of 94 randomly selected genes. The red vertical line indicates the observed value of SSMD computed for the original gene set.

We identified 31 MSigDB gene sets whose SSMD values were significantly larger than those of random control gene sets that contain the same number of genes randomly selected from all expressed genes (Bonferroni corrected *P* < 0.01, permutation test) (Table 1). These 31 gene sets, containing 1,855 distinct genes that are more likely to be aberrantly expressed in defined outlier individuals. We named these gene sets and genes L-SSMD gene sets and genes. Fig. 2 shows one of L-SSMD gene sets, *G-protein coupled receptor activity*, which contains 94 genes. In addition, eight GWAS catalog gene sets showed relatively large SSMD (*P* < 0.001, permutation test), though not significant following Bonferroni correction. These sets included genes implicated in adverse responses to chemotherapy, conduct disorder, fasting insulin-related traits, metabolite levels, obesity, retinal vascular caliber, temperament, or thyroid hormone levels (S1Table).

**Table 1:** Gene sets that tend to be aberrantly expressed in LCLs of European descent.

Gene set		# of genes
**C2: curated gene sets (Chemical and genetic perturbations, Reactome gene sets)**		
1. AIGNER_ZEB1_TARGETS	Genes up-regulated in MDA-MB-231 cells (breast cancer) after knockdown of ZEB1 [GeneID = 6935] by RNAi	28 / 35
2. CAFFAREL_RESPONSE_TO_THC_8HR_3_UP	Genes up-regulated in EVSA-T cells (breast cancer) treated with 3 micromolar THC (delta-9-tetrahydrocannabinol) [PubChem = 6610319] for 8 h.	5 / 5
3. GAUSSMANN_MLL_AF4_FUSION_TARGETS_E_UP	Up-regulated genes from the set E (Fig. 5a): specific signature shared by cells expressing either MLL-AF4 [GeneID = 4297;4299] or AF4-MLL fusion proteins alone, and those expressing both fusion proteins.	76 / 97
4. HOFMANN_MYELODYSPLASTIC_SYNDROM_RISK_UP	Genes up-regulated in bone marrow hematopoietic stem cells (HSC, CD34+ [GeneID = 947]) from patients with high risk of myelodysplastic syndrom (MDS) compared to the low risk patients.	19 / 24
5. IWANAGA_CARCINOGENESIS_BY_KRAS_UP	Cluster 3: genes up-regulated in lung tissue samples from mice with tumor-bearing genotypes (activated KRAS [GeneID = 3845] alone or together with inactivated PTEN [GeneID = 5728]).	141 / 170
6. LEIN_CHOROID_PLEXUS_MARKERS	Genes enriched in choroid plexus cells in the brain identified through correlation-based searches seeded with the choroid plexus cell-type specific gene expression patterns.	79 /103
7. LIEN_BREAST_CARCINOMA_METAPLASTIC_VS_DUCTAL_DN	Genes down-regulated between two breast carcinoma subtypes: metaplastic (MCB) and ductal (DCB).	77 / 114
8. LIU_PROSTATE_CANCER_UP	Genes up-regulated in prostate cancer samples.	79 / 96
9. MASRI_RESISTANCE_TO_TAMOXIFEN_AND_AROMATASE_INHIBITORS_UP	Genes up-regulated in derivatives of MCF-7aro cells (breast cancer) that developed resistance to tamoxifen [PubChem = 5376] or inhibitors of aromatase (CYP19A1) [GeneID = 1588].	11 / 20
10. MIKKELSEN_MEF_ICP_WITH_H3K27ME3	Genes with intermediate-CpG-density promoters (ICP) bearing the tri-methylation mark at H3K27 (H3K27me3) in MEF cells (embryonic fibroblasts).	115 / 206
11. PEPPER_CHRONIC_LYMPHOCYTIC_LEUKEMIA_DN	Genes down-regulated in CD38+ [GeneID = 952] CLL (chronic lymphocytic leukemia) cells.	11 / 21
12. POTTI_ETOPOSIDE_SENSITIVITY	Genes predicting sensitivity to etoposide [PubChem = 36462].	37 / 43
13. QI_PLASMACYTOMA_DN	Down-regulated genes that best disciminate plasmablastic plasmacytoma from plasmacytic plasmacytoma tumors.	85 / 100
14. REACTOME_CGMP_EFFECTS	Genes involved in cGMP effects	15 / 19
15. REACTOME_LIGAND_GATED_ION_CHANNEL_TRANSPORT	Genes involved in Ligand-gated ion channel transport	6 / 21
16. VANHARANTA_UTERINE_FIBROID_UP	Genes up-regulated in uterine fibroids vs normal myometrium samples.	39 / 45
17. WU_CELL_MIGRATION	Genes associated with migration rate of 40 human bladder cancer cells.	143 / 184
**C3: motif gene sets (microRNA targets)**		
18. TCCAGAG, MIR-518C	Targets of MicroRNA TCCAGAG, MIR-518C	132 / 148
**C4: computational gene sets (cancer modules, cancer gene neighborhoods)**		
19. MODULE_122	Genes in the cancer module 122	111 / 141
20. MODULE_215	Genes in the cancer module 215	3 / 15
21. MODULE_274	Genes in the cancer module 274	44 / 82
22. MORF_BCL2L11	Neighborhood of BCL2L11	123 / 188
23. MORF_MYL3	Neighborhood of MYL3	44 / 71
**C5: GO gene sets (GO biological process, GO molecular function)**		
24. EXTRACELLULAR_LIGAND_GATED_ION_CHANNEL_ACTIVITY	Genes annotated by the GO term GO:0005230. Catalysis of the transmembrane transfer of an ion by a channel that opens when a specific extracellular ligand has been bound by the channel complex or one of its constituent parts.	14 / 22
25. G_PROTEIN_COUPLED_RECEPTOR_ACTIVITY	Genes annotated by the GO term GO:0004930. A receptor that binds an extracellular ligand and transmits the signal to a heterotrimeric G-protein complex. These receptors are characteristically seven-transmembrane receptors and are made up of hetero- or homodimers.	94 / 191
26. TRANSMISSION_OF_NERVE_IMPULSE	Genes annotated by the GO term GO:0019226. The sequential electrochemical polarization and depolarization that travels across the membrane of a nerve cell (neuron) in response to stimulation.	108 / 189
**C6: oncogenic signatures**		
27. MEL18_DN.V1_DN	Genes down-regulated in DAOY cells (medulloblastoma) upon knockdown of PCGF2 [GeneID = 7703] gene by RNAi.	104 / 148
**C7: immunologic signatures**		
28. GSE19825_NAIVE_VS_DAY3_EFF_CD8_TCELL_UP	Genes up-regulated in comparison of naive CD8 T cells versus effector CD8 T cells.	128 / 200
29. GSE19825_NAIVE_VS_IL2RALOW_DAY3_EFF_CD8_TCELL_UP	Genes up-regulated in comparison of naive CD8 T cells versus effector CD8 IL2RA [GeneID = 3559] low T cells at.	133 / 200
30. GSE3982_NKCELL_VS_TH2_UP	Genes up-regulated in comparison of NK cells versus Th2 cells.	136 / 200
31. GSE8515_CTRL_VS_IL6_4H_STIM_MAC_DN	Genes down-regulated in comparison of untreated macrophages versus those treated with IL6 [GeneID = 3569].	144 / 200

The names of gene sets and MSigDB subclasses are given. Number (#) of genes shows the number of genes included in SSMD computation and the number of genes in the original gene set.

### Outlier individuals in L-SSMD gene sets

To identify outlier individuals, we applied chi-square plot to examine MD values of all individuals with respect to each of the 31 L-SSMD gene sets. We identified 17 distinct outliers in total, 11 of which were found in more than one gene set, and almost all gene sets had more than one outlier. The distributions of outliers in the gene sets are given in S1 Fig Fig. 2 shows that three outliers were detected in the L-SSMD gene set, G-protein coupled receptor activity, using chi-square plot.

### Gene sets (S-SSMD) that tend not to be aberrantly expressed

Fourteen gene sets with significantly smaller SSMD (S-SSMD) were identified (Bonferroni corrected *P* < 0.01, Table 2). The S-SSMD genes (*n* = 534) in the 14 S-SSMD gene sets are involved in homologous recombination repair of replication-independent double-strand breaks, catalysis of the transfer of a phosphate group to a carbohydrate substrate molecule, or cell cycle control. GWAS gene sets implicated in alcohol dependence and metabolic syndrome showed significantly smaller SSMD than random gene set (S1 Table).

**Table 2:** Gene sets that tend not to be aberrantly expressed in LCLs of European descent.

Gene set		# of genes
**C2: curated gene sets (Canonical pathways, KEGG, Reactome, BioCarta, chemical and genetic perturbations)**		
1. PID_ATM_PATHWAY	ATM pathway	34 / 34
2. KEGG_HOMOLOGOUS_RECOMBINATION	Homologous recombination	28 / 28
3. MORI_PRE_BI_LYMPHOCYTE_DN	Down-regulated genes in the B lymphocyte developmental signature, based on expression profiling of lymphomas from the Emu-myc transgenic mice: the Pre-BI stage.	73 / 77
4. XU_RESPONSE_TO_TRETINOIN_UP	Genes up-regulated in NB4 cells (acute promyelocytic leukemia, APL) by tretinoinalone.	14 / 16
5. FLECHNER_PBL_KIDNEY_TRANSPLANT_OK_VS_DONOR_DN	Genes downregulated in peripheral blood lymphocytes (PBL) from patients with well functioning kidneys more than 1-year post transplant compared to those from normal living kidney donors	40 / 41
6. GARGALOVIC_RESPONSE_TO_OXIDIZED_PHOSPHOLIPIDS_LIGHTYELLOW_UP	Genes from the lightyellow module which are up-regulated in HAEC cells (primary aortic endothelium) after exposure to the oxidized 1-palmitoyl-2-arachidonyl-sn-3-glycerophosphorylcholine (oxPAPC).	11 / 11
7. REACTOME_HOMOLOGOUS_RECOMBINATION_REPAIR_OF_REPLICATION_INDEPENDENT_DOUBLE_STRAND_BREAKS	Genes involved in Homologous recombination repair of replication-independent double-strand breaks	16 / 17
8. REACTOME_G1_PHASE	Genes involved in G1 Phase.	35 / 38
9. BIOCARTA_ATRBRCA_PATHWAY	Role of BRCA1, BRCA2 and ATR in Cancer Susceptibility.	21 / 21
**C4: computational gene sets (cancer modules, cancer gene neighborhoods)**		
10. MODULE_87	Genes in the cancer module 87	44 / 44
11. MORF_PRKAR1A	Neighborhood of PRKAR1A protein kinase, cAMP-dependent, regulatory, type I, alpha (tissue specific extinguisher 1) in the MORF expression compendium	139 / 142
12. MORF_REV3L	Neighborhood of REV3L	55 / 57
13. GNF2_DDX5	Neighborhood of DDX5 DEAD (Asp-Glu-Ala-Asp) box polypeptide 5 in the GNF2 expression compendium	62 / 63
**C5: GO gene sets (GO molecular function)**		
14. CARBOHYDRATE_KINASE_ACTIVITY	Genes annotated by the GO term GO:0019200. Catalysis of the transfer of a phosphate group, usually from ATP, to a carbohydrate substrate molecule.	15 / 15

### Validation of L- and S-SSMD gene sets

**Figure 3:**
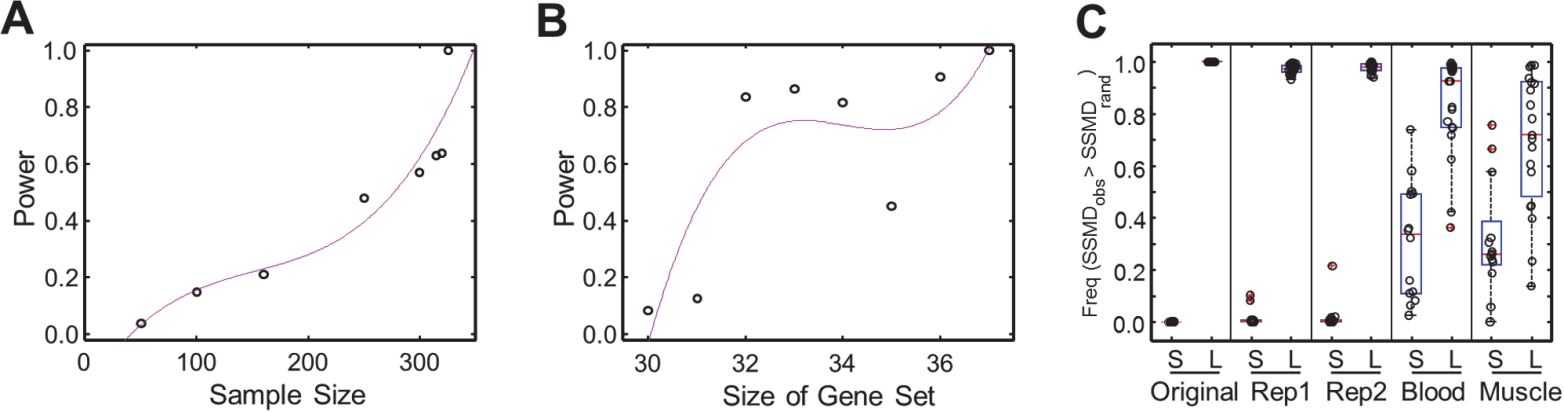
Power of SSMD test and validation of significant L- and S-SSMD gene sets. (**A**) The change of power as a function of sample size. (**B**) The change of power as a function of the size of a gene set. (**C**) Validation of significant L- and S-SSMD gene sets using different expression data. Original: Geuvadis LCL expression data normalized using PEER (i.e., data used for the main results); Rep1: first set of replication of Geuvadis LCL expression data without PEER normalization; Rep2: second set of replication of Geuvadis LCL expression data without PEER normalization; Whole blood: GTEx whole blood expression data; and Muscle: GTEx muscle expression data. The boxplot shows the frequency of observed SSMD is greater than the control SSMD of 1,000 random replicates.

We evaluated the power of SSMD as a statistic describing the propensity of a gene set for aberrant expression. We considered the influences of the sample size (*n*) and the size of gene set (*m*). In cases where the SSMD are insensitive to *n* or *m*, the power would be maintained when *n* or *m* changes. However, we found that the power dropped substantially when *n* dropped from 326 to 300 or when *m* dropped from 37 to 31, suggesting that SSMD is sensitive to both *n* and *m* (Fig. 3A, B). This might be due to that only a small number of genes in the gene set tested that were expressed aberrantly in few individuals, and the power analyses for *m* and *n* were based on the sub-sampling of genes and individual samples, respectively (Materials and Methods).

Nevertheless, owning to the sensitivities, it was necessary to validate our results of identified L- and S-SSMD gene sets, which were obtained using the Geuvadis LCL expression data [3]. We validated our results by taking into consideration three factors: (1) the robustness against the influence of data normalization methods, (2) the replicability against technical variability, and (3) the reproducibility against independent expression data of different tissues.

The “original” Geuvadis expression data we used to identify L- and S-SSMD gene sets had been normalized by using the algorithm of probabilistic estimation of expression residuals (PEER) [16, 17]. We first showed that the PEER normalization algorithm did not change our results. To do so, we downloaded the “raw” Geuvadis expression data quantified in reads per kilobase per million (RPKM) without PEER normalization. Two replicate sets of raw RPKM data were available for most of the Geuvadis samples. We therefore used each set independently to test the significance of SSMD for L- and S-SSMD gene sets against random control sets. The procedure was similar to what we used for establishing the original L- and S-SSMD gene sets. Briefly, for each L- or S-SSMD gene set, we tested whether the SSMD computed with raw RPKM data tended to be larger or smaller than that of random gene sets. The observed SSMD was compared against SSMD values computed from 1,000 replicates of randomly selected genes and the significance was evaluated by examining how many times the observed SSMD was larger or smaller than random SSMD. As expected, with the original (PEER normalized) expression data, all 31 L-SSMD gene sets had a larger SSMD than sets of randomly selected genes, while all 14 S-SSMD gene sets had a smaller SSMD. The same patterns were recovered with the raw RPKM expression data (Fig. 3C). These results indicated that our results for L- or S-SSMD gene sets were robust against the normalization methods and the technical variability.

In addition, we used independent gene expression data from tissues different from LCL to validate our results. We obtained the expression data of whole blood and muscle (in 156 and 138 samples, respectively) from the pilot study of the Genotype-Tissue Expression project (GTEx) [18]. We re-computed SSMD using the GTEx data and conducted the same validation tests. With GTEx data, the frequency of observed SSMD greater than random SSMD was significantly higher for L-SSMD gene sets than S-SSMD gene sets (Kolmogorov-Smirnov [K-S] test, *P* = 1.02e-5 and 9.9e-4, for whole blood and muscle, respectively, Fig. 3C). These results suggested that gene sets tending to have larger observed SSMD in LCL were more likely to have larger SSMD in the other two tested tissues, or vice versa. The consistency in the direction of SSMD patterns validates the biological significance of L- and S-SSMD gene sets.

### Differences in aberrant expression between Europeans and Africans

Next we examined which gene sets show strong population-specific SSMD. For a given gene set, we first computed MD_i_ with the gene expression data for all 402 samples of both European (EUR, *n* = 326) and African (AFR, *n* = 76) ancestries. We then use these MD_i_ to compute *SSMD*_EUR_ and *SSMD*_AFR_ for EUR and AFR samples, respectively, and calculated the difference in SSMD between them: *diffSSMD*_EUR-YRI_ = *SSMD*_EUR_-*SSMD*_AFR_. To assess the significance, we computed *diffSSMD*_rand_ by randomly assigning samples without regard to their identities of original populations. For each gene set, we computed 1,000 permutations of *diffSSMD*_rand_ to obtain the null distribution of expected *diffSSMD*_EUR-YRI_. We compared the value of *diffSSMD*_EUR-YRI_ with the null distribution to obtain its significance.

**Figure 4:**
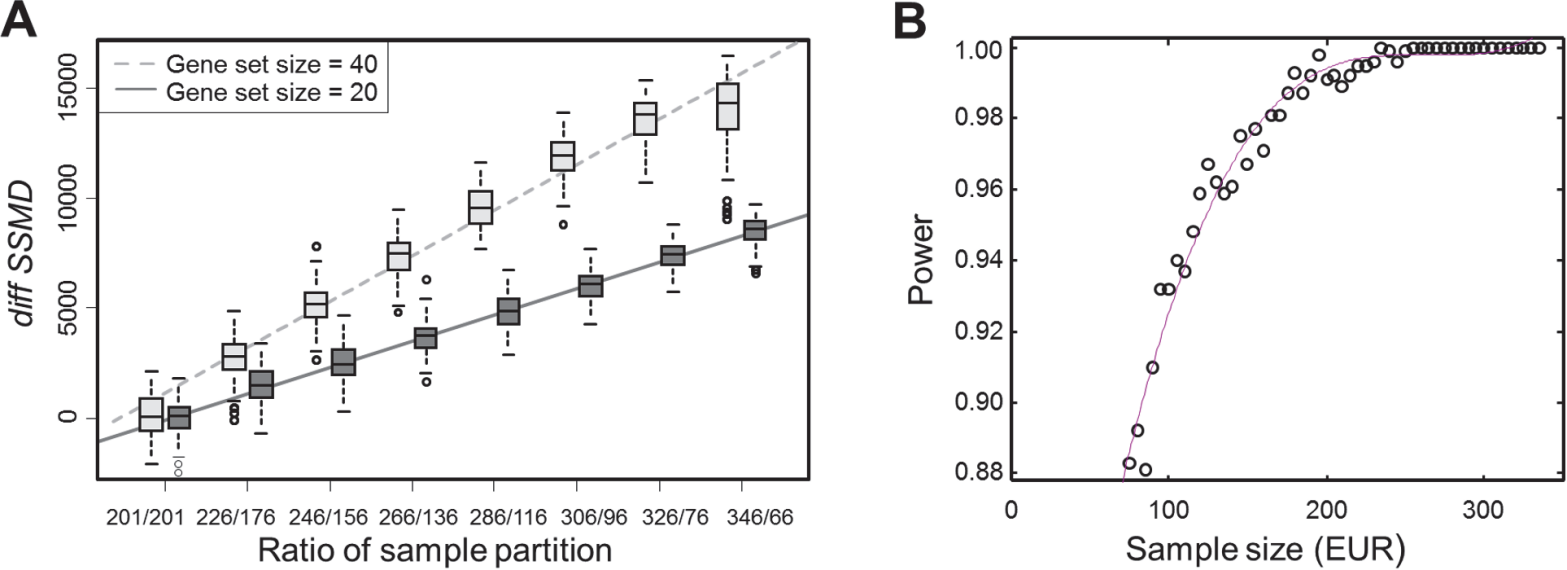
Change of *diffSSMD* as a function of the ratio between partitioned samples and the power of *diffSSMD* test under varying sample size. (**A**) The change of *diffSSMD* as a function of the size ratio of partitioned samples. The results with respect to two gene sets of size 20 and 40 are shown. For each ratio of partition, the distribution of *diffSSMD*_rand_ were constructed from 100 randomly shuffled samples. (**B**) The change of the power of the *diffSSMD* test between EUR and AFR populations for the population-specific effect as a function of the size of EUR samples. The red line is fitted by using polynomial regression with the cubic model.

We used two random sets of genes (*n* = 20 and 40) to show that the values of *diffSSMD* were proportional to gene set size and changed linearly with the ratio by which the total samples were partitioned into two sub-groups (Fig. 4A). In the test, we ignored the EUR and AFR ancestries of samples. We randomly shuffled the 402 samples, partitioned them to two sub-groups with different ratios (such as, 201/201 or 326/76), and computed the *diffSSMD* between the two sub-groups. We repeated this 1,000 times per ratio to obtain null distributions of *diffSSMD*. We found that, regardless of gene set size, when samples were partitioned into groups of equal size (i.e., 201/201), the average *diffSSMD* was close to zero. When samples were partitioned unequally, the average value of *diffSSMD* increased with the degree of inequality in a linear manner. When the ratio of partition was fixed (e.g., 326/76, the actual sample ratio of EUR and AFR), the average *diffSSMD* reflected the size of the gene set (e.g., twice as large for the 40-gene set as the 20-gene set). When both the ratio of partition and the gene set was fixed, as we did in the real test for each gene set, the values of null *diffSSMD* fluctuated only due to the random assignment of samples into the two sub-groups. Similarly, in our significance test for *diffSSMD*_EUR-YRI_, both the gene set size and the ratio of partition (=326/76) were fixed, and the null distribution of *diffSSMD*, *diffSSMD*_rand_, was constructed from 1,000 random repeats of the partition of shuffled samples. An observed *diffSSMD*_EUR-YRI_ was considered to be significant when it was greater or smaller than all values of *diffSSMD*_rand_.

In total, 231 gene sets showed significantly smaller *diffSSMD*_EUR-YRI_ than *diffSSMD*_rand_ in our analysis (S2 Table). For these gene sets, the differences between *SSMD*_EUR_ and *SSMD*_AFR_ were relatively smaller than those differences calculated when EUR and AFR individuals were randomly assigned. This was likely caused by the relatively large *SSMD*_AFR_ in real data. In other words, AFR samples were more likely to produce disproportionally larger SSMD than EUR samples.

In contrast, only four gene sets showed the opposite pattern—that is, for these genes, *diffSSMD*_EUR-YRI_ was significantly larger than *diffSSMD*_rand_. Genes in these four sets included: (1) genes involved in the process preventing the degeneration of the photoreceptor (a specialized cell type that is sensitive to light), (2) genes down-regulated in prostate tumor (a tumor with distinct signatures differentiate between African-American and European-American patients [19]), (3) genes associated with malignant fibrous histiocytoma tumors, and (4) genes up-regulated in colon tissue upon the knockout of *MBD2*, a methyl-CpG binding protein that mediates the methylation signal.

Finally, the power analysis for *diffSSMD*_EUR-YRI_ was conducted using the first gene set among the four with significantly larger *diffSSMD*_EUR-YRI_. The result suggested that the difference in sample size between EUR and AFR had little impact on the sensitivity of asserting that the tested gene set was significant. As shown in Fig. 4B, when the EUR were subsampled from 326 to 76 (the sample size of AFR), the power of *diffSSMD* only slightly decreased.

### Genetic and non-genetic factors contributing to aberrant expression

To evaluate the contributions of genetic or non-genetic factors in causing aberrant expression, we utilized three statistical metrics to characterize L- and S-SSMD genes and compared the properties of the two groups of genes (Materials and Methods). The three metrics are: (1) the discordant gene expression, measured as the relative mean difference in gene expression, between twin pairs, considering both monozygotic (MZ) and dizygotic (DZ) twins [9]; (2) the narrow-sense heritability (*h* ^2^) of gene expression [20]; and (3) the coefficient of variation (CV) of single-cell gene expression [21].

**Figure 5:**
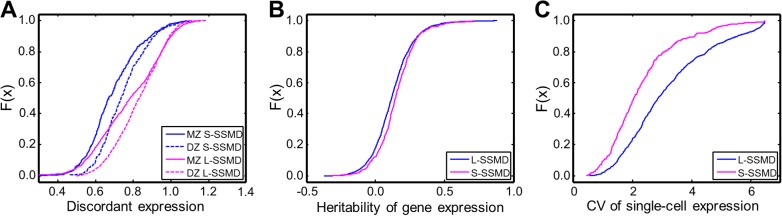
Differences in expression discordance, heritability and variability between L- and S-SSMD genes. (**A**) Normalized mean discordant expression (measure as the relative mean difference, RMD) per gene. (**B**) Heritability of gene expression. (**C**) Coefficient of variation of single-cell expression.

The discordant expression between twin pairs in L-SSMD genes is greater than that in S-SSMD genes (*P* = 2.8e-15 between MZ pairs and 3.0e-34 between DZ pairs; K-S test, Fig. 5A). The more pronounced discordant expression between MZ pairs for L-SSMD genes, compared to S-SSMD genes, is likely due to the effect of environmental factors. L-SSMD genes may have increased sensitivity to environmental factors. On the other hand, regardless of L- or S-SSMD genes, the discordant expression is always greater between DZ pairs than between MZ pairs. This suggests that genetic diversity increases the level of discordance in gene expression. The difference is more pronounced for L-SSMD genes (*P* = 5.6e-23 and 5.4e-6 for L- and S-SSMD genes, respectively; S3 Table).

L-SSMD genes tend to have a smaller *h* ^2^ than S-SSMD genes (*P* = 3.6e-5, K-S test, Fig. 5B). Similar results were obtained with different *h* ^2^ estimates (e.g., those using data from another twin cohort [22] and those using data from unrelated individuals [23]). Furthermore, L-SSMD genes showed greater expression variability at the single-cell level than S-SSMD (*P* = 7.7e-21, K-S test, Fig. 5C). Forty genes were found to be shared between L-SSMD and S-SSMD groups. Excluding these overlapping genes did not qualitatively change any results described above.

### Common regulatory variation is not responsible for aberrant expression

To evaluate the contribution of eQTLs to aberrant expression, we obtained 419,983 *cis*-acting eQTL SNPs (eSNPs) associated with 13,703 genes from a previous study [3]. We found that 20.3% of L-SSMD genes and 19.3% of S-SSMD genes have *cis*-eSNP(s). That is to say, there is no difference in *cis*-eSNP existence between L- and S-SSMD genes (*P* = 0.67, Fisher’s exact test). Due to the prevalence of eSNPs, this result was not unexpected.

Next we set out to examine whether outlier individuals are more likely to have an eQTL genotype that might explain their outlier status. In particular, we calculated the genotype-scaled effect size (**β** = |β|*genotype, where genotype = {0,1,2}, to take into account of the direction of the effect) for all *cis*-eSNPs of associated genes in L-SSMD gene sets for outlier individuals. Multiple eSNPs in the same genes were treated independently and the values of genotype-scaled effect sizes calculated were pooled together as **β**_outlier_. We did the same calculation for the same sets of genes for all non-outlier individuals and obtained **β**_non-outlier_.

**Figure 6:**
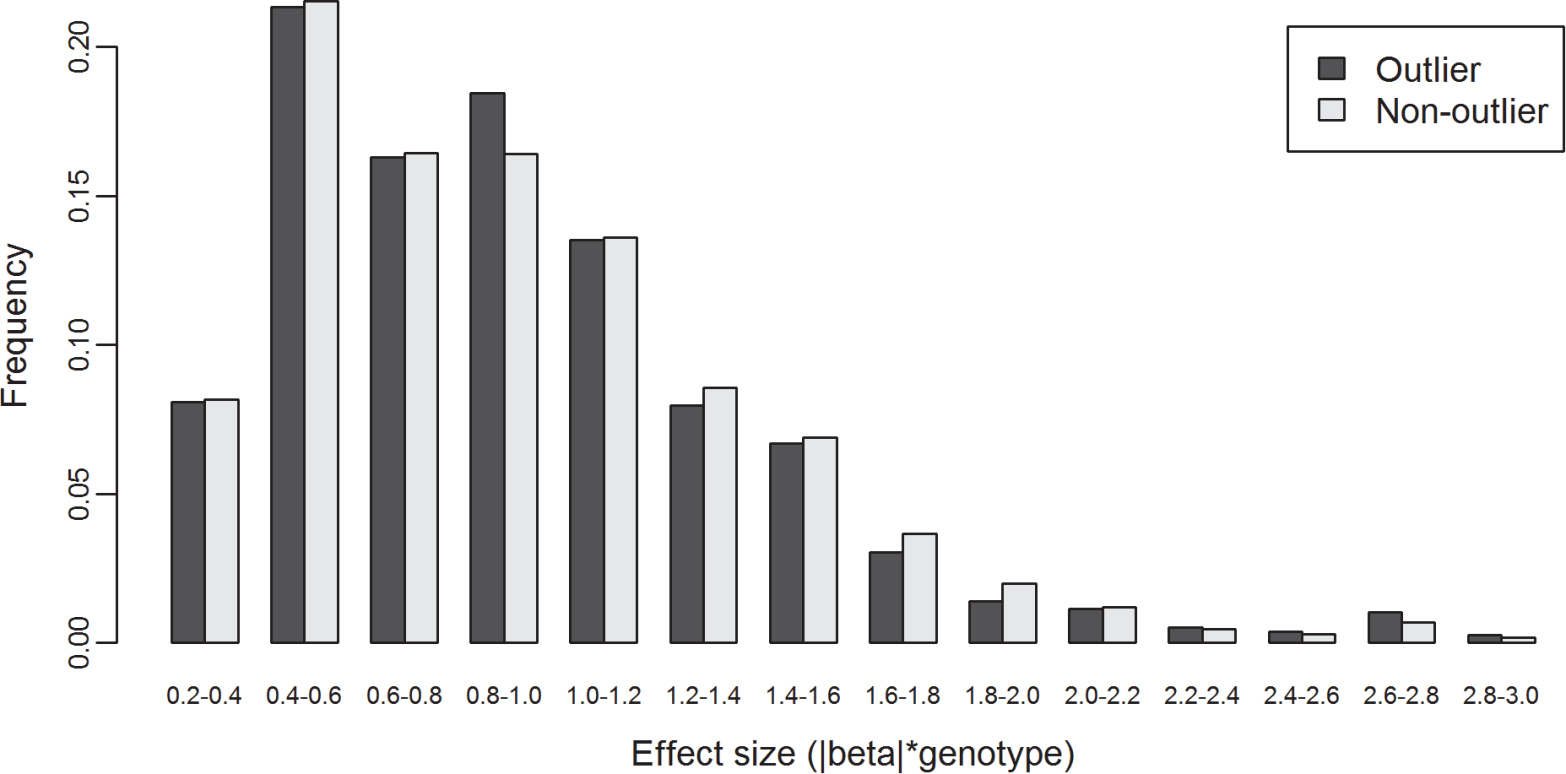
Distributions of nonzero effect size β of *cis*-eSNPs of L-SSMD genes in outlier and non-outlier individuals. The effect size β is genotype-weighted (i.e., β =|β|*genotype, where genotype={0,1,2}).

We hypothesized that if *cis*-eSNPs cause the outlier’s gene expression level to deviate away from the population mean, then the genotype-scaled effect size of these eSNPs in outlier individuals should be less likely to be zero and more likely to be larger than that of non-outlier individuals. However, we found that 45.3% of **β**_outlier_ (*n* = 24,649, pooling from 63 outlier-gene pairs, i.e., pairs of outlier individual and gene in corresponding gene sets) and 46.2% of **β**_non-outlier_ (*n* = 3,329,296, pooling from 309 outlier-gene pairs) were zeros. There was no difference between the two fractions (*P* = 0.086, χ^2^ test). Considering that this result might be affected by the uncontrolled linkage disequilibrium between eSNPs, we re-performed the analysis using only the most significant eSNP per gene. With such a single-eSNP setting, we found that 9.49% of **β**_outlier_ (*n* = 875, pooling from 63 outlier-gene pairs) and 10.58% of **β**_non-outlier_ (*n* = 118,965, pooling from 309 outlier-gene pairs) were zeros. Again, there was no difference between the two fractions (*P* = 0.3448, χ^2^ test). Furthermore, with only the most significant *cis*-eSNP per gene, we found that the distribution of nonzero **β**_outlier_ was similar to that of nonzero **β**_non-outlier_ (K-S test, *P* = 0.67, Fig. 6).

These results suggest that eSNPs, as commonly-occurring regulatory genetic variants, may not be responsible for aberrant expression of genes under their regulation.

### Private variants may be responsible for aberrant expression

We resorted to examining whether private SNPs are responsible for aberrant expression. We tested whether private SNPs are enriched in regulatory regions of L-SSMD genes in outlier individuals. The SNP density was calculated by pooling SNPs, which are private to each outlier individual, in 1Mb *cis*-regulatory regions of L-SSMD genes. Based on the ENCODE annotations [24], the regulatory regions were divided into seven subclasses, namely, E (predicted enhancer), TSS (predicted promoter region including TSS), T (predicted transcribed region), PF (predicted promoter flanking region), CTCF (CTCF-enriched element), R (predicted repressed or low-activity region), and WE (predicted weak enhancer or open chromatin *cis*-regulatory element).

We found that the density of private SNPs in E regions of L-SSMD genes in outlier individuals was significantly higher than that in the same E regions in non-outlier individuals (*P* < 0.001, one-tailed *t* test). The density was also significantly higher than that derived from three additional control settings, including the reconstructed E regions from the locations 10 Mb away from genes, and randomly selected L-SSMD or S-SSMD genes (Materials and Methods). In summary, we randomly selected individuals or genes in a total of four different manners to construct the control scenario, from which the private SNP density was calculated and compared with the observed density. The most salient finding was that for the E regions, the observed density of private SNPs in L-SSMD genes was significantly higher than any of the controls (Table 3). In addition, we also found that, for TSS, the density is significantly higher than three controls (*P* < 0.001, one-tailed *t* test). These results are consistent with the findings of a previous study, which also focused on the effects of rare variant on causing outlier expression [25]. The rest of the region classes showed less significant enrichment or similar levels of the density (Table 3). For illustrative purpose, two private SNPs, rs189458147 and rs117086221, located in E region of *PMAIP1* and TSS region of *NEIL1* are depicted (S2 Fig).

**Table 3:** Density of private SNPs in ENCODE regulatory regions of L-SSMD genes.

		Density of private SNP (per million bp)				
Abbreviation	Description	Observed (#/Mb)	Control 1	Control 2	Control 3	Control 4
E	Predicted enhancer	2.07 (308/149)	1.54*	1.41*	1.76*	1.73*
TSS	Predicted promoter region including transcription start site	1.91 (408/214)	1.51*	1.23*	1.45*	1.82
CTCF	CTCF enriched element	1.89 (213/113)	1.71*	1.34*	1.56*	1.79
T	Predicted transcribed region	2.00 (4184/2092)	1.79*	1.52*	1.83	1.92
PF	Predicted promoter flanking region	1.79 (94/53)	1.46*	1.33*	1.69	1.96
R	Predicted repressed or low activity region	1.88 (10152/5400)	1.72*	1.45*	1.68*	1.79
WE	Predicted weak enhancer or open chromatin *cis* regulatory element	1.93 (102/53)	1.59*	1.63*	2.15	2.00
UNCL	Unclassified region	1.64 (833/508)	1.41*	0.84*	1.53	1.60

The symbol * indicates the SNP density in the corresponding control regions is significantly lower than that in the test regions of the outlier. The significance is assessed by one-tailed t test at the level of *P* = 0.001. Control 1: randomly selected non-outlier individuals to replace outlier individuals. Control 2: randomly selected genomic region that locate 10 Mb away from L-SSMD genes. Control 3: select randomly shuffled L-SSMD genes to the same amount of original gene set. Control 4: select randomly shuffled S-SSMD genes to the same amount of original gene set.

## Discussion

We have used MD as a measure of distance between two points in the space defined by two or more correlated variables to quantify the deviation of individuals’ gene-set expression to the population mean. This quantity allowed us to identify outliers. The sum of the quantity across individuals (i.e., SSMD) allowed us to assess how likely a gene set is to be aberrantly expressed in outlier individuals. As expected, genes involved in fundamental molecular functions and metabolic pathways are unlikely to be aberrantly expressed, showing a small SSMD. In contrast, genes in the gene sets with large SSMD tend to be involved in regulation of cellular processes and modulation of signal transduction (see Table 1). Notably, three gene sets with large SSMD have GO definitions: (1) extracellular ligand gated ion channel activity, (2) G-protein coupled receptor activity, and (3) transmission of nerve impulse. G-protein coupled receptors constitute a large protein family of receptors that sense molecules outside the cell and activate inside signal transduction pathways, implicated in various human diseases and development processes [26–28].

Widespread genetic regulatory variants have been uncovered by eQTL analyses. Most eQTLs are detected based on linear regression between genotype and gene expression level. The inherent limitation of this method is that only commonly-occurring regulatory genetic variants will be discovered. Our analysis of *cis*-acting eQTLs in gene sets suggests that the observed patterns of expression are unlikely to be related to commonly-occurring regulatory genetic variation. The fact that eQTLs are less likely to be responsible for aberrant expression of genes under their regulation underscores the technical limitation of the eQTL method in dealing with gene expression regulation in outliers.

Instead we discovered that private SNPs are likely to be responsible for aberrant expression. Our results suggest that private SNPs are significantly enriched in enhancer and promoter regions of aberrantly-expressed genes. This is in agreement with the findings of [25], in which Montgomery and colleagues reported the identification of the signal of rare SNPs underlying large changes in gene expression by calculating whether individuals with outlier array expression values are enriched for rare genetic variants. They used Z-score as a measurement of how far the observed value is from the mean of the sample. They found that individuals with gene expression Z-score ≥ 2 have an excess of rare variants within 100 kb of the transcription start site. The signal was found to be statistically significant for rare variants landing in highly conserved sites [25]. Taken together, results from both studies suggest that rare or private SNPs contribute to the large changes in gene expression. Awareness of this effect is important as it means that a rare genetic variant, even only seen in an individual genome, could potentially be regulating the expression of the phenotype to an extreme extent relative to the population mean. This makes sense because the recent explosion of human population size has created abundances of rare variants [29]. These variants, segregating in single individuals or only in small groups of people, have not been subject to the test of natural selection, and thus can potentially have stronger functional consequences. They may underlie aberrant gene expression and may also underlie susceptibility to complex diseases. Therefore, the individual bearing private SNPs causing aberrant gene expression might be an interesting model of phenotypes relevant to the function of the aberrantly-expressed gene. Otherwise, on the population level, the variants may bear little relevance to the phenotypes.

Intrinsic properties of gene sets are defined not only by descriptive functions of genes they include but also several measurable genetic metrics. Combined use of these metrics has demonstrated the contribution of both genetic and environmental factors to aberrant expression. First, twin data facilitated the dissection of the contributions of genetic and non-genetic factors. The discordance in gene expression is expected to be larger between pairs of dizygotic (DZ) twins than between pairs of monozygotic (MZ) twins, as the phenotypic difference between DZ pairs may result from both genetic and environmental effects. We indeed observed the difference between MZ and DZ in discordant expression as expected, and to the same extent for both genes tending to and tending not to be aberrantly expressed. This result suggests that genetic diversity increases overall expression variability. More importantly, we found that the discordant expression in MZ pairs for genes tending to be aberrantly expressed is greater than that for genes that tend not to be aberrantly expressed. This result suggests that under the same genetic background, aberrantly expressed genes are more likely to be sensitive to the change of environmental factors than non-aberrantly expressed genes. Second, heritability is a dimensionless measure of the weight of genetic factors in explaining the phenotypic variation among individuals [30–32]. We showed that genes with small SSMD have a higher narrow-sense heritability of gene expression than genes with large SSMD. Third, we detected that genes tending to be aberrantly expressed have a higher expression variability at the single-cell level than genes tending not to be aberrantly expressed. This result suggests that intrinsic single-cell expression contributes to aberrant expression.

In summary, we leveraged the 1,000 genomes RNA-seq data to identify aberrant gene expression in humans, and described a multivariate framework for detecting aberrantly-expressed gene sets and outlier individuals, offering a new way of measuring inter-individual variation in gene expression. This novel perspective on how to measure differences in gene expression between individual human subjects may provide important clues into the mechanisms of human adaptation, and may also be helpful for the arising field of personalized medicine.

## Materials and Methods

### Geuvadis RNA-seq data

We downloaded gene expression data produced by the Geuvadis project RNA-seq study [3] from the website of EBI ArrayExpress via accessions E-GEUV-1 and E-GEUV-3. The samples included 462 unrelated human LCLs from the EUR (CEU, FIN, GBR, TSI) and YRI populations, most of which had been sequenced in the 1000 Genome Project Phase 1. The expression data were normalized by using the algorithm of probabilistic estimation of expression residuals (PEER) [3, 17, 33]. To minimize the impact of unspecific sources on measurement of individual’s expression, principal component analysis (PCA) was applied to the full expression matrix. Based on the PCA results, 19 EUR individuals with unusual global expression profiles relative to the rest of individuals in the population were excluded due to potential technical artifacts (S3 Fig). We also excluded individuals whose genotype information was unavailable in the 1000 Genome Project Phase 1, resulting in a total of 402 remaining samples (326 EUR and 76 AFR).

### Annotated gene sets

Gene sets were downloaded from MSigDB v4.0 [14]. The MSigDB gene sets had been divided into seven groups: C1—positional gene sets (*n* = 326), C2—Curated gene set (*n* = 4,722), C3—motif gene (*n* = 836), C4—Computational gene sets (*n* = 858), C5— GO gene sets (*n* = 1,454), C6—oncogenic signatures (*n* = 189), and C7—immunologic signatures (*n* = 1,910). The annotated gene sets of the NHGRI GWAS Catalog [15] were obtained from http://www.genome.gov/gwastudies (accessed April 2014).

### Robust MD calculation

To calculate MD, the correlation between the expression profiles of individuals was captured by the *inter-individual expression covariance*, *Cov*_ab_. For expression *E* between any two individuals *a* and *b*, *Cov*_ab_ is computed as:

$$Cov_{ab}=\frac{\sum_{k=1}^{m}\left(E_{ak}-\mu_{a})(E_{bk}-\mu_{b}\right)}{m-1},$$

where *m* is the number of genes in the gene set under study, and *µ_a_* and *µ_b_* are the mean gene expression values for individuals *a* and *b*, respectively. Given all pair-wise comparisons of individuals we obtained the inter-individual covariance matrix *Cov*. We employed the minimum covariance determinant (MCD) estimator [34] to compute a robust version of *Cov*, as implemented in the Matlab toolbox LIBRA [35]. We then computed the MD for each individual as

$$MD_{i}=\sqrt{{\left(E_{i\cdot }-\vec{\mu }\right)}^{T}Cov^{-1}(E_{i\cdot }-\vec{\mu })},$$

where $\vec{\mu }$is *m* length vector of the per-gene mean values across all individuals.

The statistic $SSMD=\sum MD_{i}^{2}$was calculated for each set. To approximate the empirical null distributions for SSMD, we applied resampling for gene sets with different numbers of genes, ranging from 2 to 150. For a given number of genes *m*, we randomly sampled *m* genes from the full expression matrix without replacement, and then computed SSMD for the resampled gene set. The procedure was repeated 1,000 times for all gene sets. More permutations were performed for significant gene sets until the desired Bonferroni correction level *P* = 0.01 was either achieved or rejected. The resampling process breaks correlation structure between genes, hence providing a background distribution of expected random distribution of SSMD. We compared the SSMD in the observed gene set to equally-sized sets drawn at random from all assayed genes.

The chi-square plot was plotted as the ***I*** ranked MD value against the values of χ^2^(*p*, *m*), where *p* = (*i*-0.5)/***I*** and *m* is the number of genes in the gene set. The right panel of Fig. 1 is the chi-square plot that supports the multivariate outliers identified [13]. A chi-square plot draws the empirical distribution function of the square of the MD against the χ^2^ distribution with degree of freedom equal to *m*. A break in the tail of the χ^2^ distribution is an indicator for outliers [36], given that the square of the MD is approximately distributed as a χ^2^ distribution [13, 37].

### Power analysis for SSMD test

To evaluate the sensitivity of SSMD as a statistic for detecting L-SSMD gene set, power analyses were conducted. One selected L-SSMD gene set, POTTI_ETOPOSIDE_SENSITIVITY, was used as the test set. The impacts of sample size (*n*) and the size of gene set (*m*) were considered. The selected L-SSMD gene set contained 37 genes, that is, *m* = 37, while the sample size *n* = 326. The original expression data matrix was subsampled by lowering either *n* or *m*. For each subsampled *n* or *m* value, 100 random replicates of expression data matrix were constructed. The SSMD was computed for each subsampled replicate and the significance of the observed SSMD was assessed by permutation tests, as described above for detecting L-SSMD gene sets. The more sensitive is SSMD to *n* or *m*, the less would subsampled replicates remain significant as an L-SSMD.

### Discordant expression, heritability, and single-cell gene expression

To compute the discordant expression of genes between twin pairs, twinsUK gene expression data from the study of [22] were acquired. The discordant expression, i.e., the expression differences between each pair of twins, was measured as done previously [9]. Briefly, for each gene, the relative mean difference (RMD) in expression between MZ twin pairs and between DZ twin pairs was computed. For a pair of MZ twins, *i*, for example, the RMD was computed using $RMD_{i}=\frac{\left|y_{i}^{MZ1}-y_{i}^{MZ2}\right|}{2{\bar{y}}_{i}}$, where ${\bar{y}}_{i}$ is the arithmetic mean of the levels of gene expression for that MZ twin pair (designated as $y_{i}^{MZ1}$ and $y_{i}^{MZ2}$). For each gene, the data from all MZ or DZ twin pairs were pooled to compute the mean RMD per gene, $\frac{1}{n}\sum RMD_{i}$, where *n* is the number of twin pairs. The computed mean RMD per gene was normalized by the value computed in the same way but with the expression data reconstructed by randomly assigning the identities of twin pairs. The values of narrow-sense heritability (*h* ^2^) of gene expression were obtained from the study of [20]. The different estimates of *h* ^2^ were also obtained from the studies of [22] and [23]. The single-cell gene expression levels measured in 42 LCLs were acquired from the study of [21].

### Effect size of common eSNPs

The absolute value of slope coefficient (|β|) of the linear regression model was used as the measure of the effect size of each eSNP. The gene expression levels across individuals were normalized using Z-score to make the values of β uncorrelated with the total gene expression levels. The sign of β was ignored because it is only relative against the genotypes of each eSNP, which were denoted by 0 for homozygous major alleles, 1 for heterozygous alleles, and 2 for homozygous minor alleles. Instead, an eSNP’s effect direction was determined by whether the eSNP causes gene expression to shift away from or towards the mean gene expression for the majority of individuals in the populations. In this sense, the notation of genotypes (0,1,2) provided the information of effect direction for eSNP. If an individual’s eSNP genotype is 0, then the effect of the eSNP is to maintain the same expression level for the eSNP-regulated gene between outlier individuals and the majority of individuals in the population; on the other hand, if the eSNP’s genotype is 1 or 2, then the effect of the eSNP is to either increase or decrease (depending on the sign of the slope) the expression of the gene by one or two times of |β| than that of genotype 0. Therefore, the effect size was weighted by the genotype: **β** = |β|*genotype. The genotype-scaled effect size was used in the comparison of the combined eSNP effects between outlier and non-outlier individuals.

### Density of private SNPs in regulatory regions of L-SSMD genes

Both heterozygous and homozygous private SNPs, with allele frequency of 1/(2*N*) and 1/*N*, respectively, for each individual (where *N* is the number of individuals), were counted. The *cis*-regions of tested genes were split into seven subclasses of regulatory regions, according to the combined chromatin state segmentation of the ENCODE GM12878 sample [24]. The density of private SNPs in each subclass of the regions was assessed for enrichment significance by comparing the observed density with that of randomly generated control regions. To provide comprehensive controls, four different means were used to construct control regions: (1) randomly selected non-outlier individuals to replace outlier individuals, (2) randomly selected genomic regions located 10 Mb away from L-SSMD genes, (3) randomly selected shuffled L-SSMD genes in the same amount of original gene set, and (4) shuffled S-SSMD genes in the same amount of original gene sets.

## Supporting Information

S1 Fig Distribution of outliers in corresponding gene sets. The 63 outliers (involving 17 distinct individuals) with respect to the 31 L-SSMD gene sets, detected by using chi-square plot, are highlighted with shaded box. The indexes of L-SSMD gene sets are given and their names are given in Table 1 of the main text.
(PDF)

S2 Fig Private SNPs located in ENCODE E (predicted enhancer) and TSS (predicted transcribed region) regions of corresponding L-SSMD genes. **(A)** Rs117086221 is located in the TSS region of gene *NEIL1* in the individual NA12154. **(B)** Rs189458147 locates in the potential E region of gene *PMAIP1* in the individual HG00122.
(PDF)

S3 Fig PCA with global gene expression data assists the removal of outliers. **(A)** A total of 19 outliers removed. They are: HG00099, HG00329, HG00125, NA12004, NA07051, HG00358, NA12399, HG00280, NA20502, NA07346, NA20792, NA12340, NA12716, NA12342, NA12842, NA20785, NA12044, NA12058, and NA07347 from populations of GBR, FIN, GBR, CEU, CEU, FIN, CEU, FIN, TSI, CEU, TSI, CEU, CEU, CEU, CEU, TSI, CEU, CEU, and CEU, respectively. **(B)** PCA result after the outliers are removed.
(PDF)

S1 Table GWAS gene sets that tend to be aberrantly expressed in LCLs of European descent. (PDF)

S2 Table Gene sets with significant *diffSSMD = SSMD_EUR_-SSMD_YRI_*. (PDF)

S3 Table *P*-values of Kolmogorov-Smirnov test for the normalized relative mean difference (RMD) between “L-SSMD” and “S-SSMD” genes in monozygotic (MZ) and dizygotic (DZ) twins. (PDF)
