# Supplementary figures and images for: Aberrant Gene Expression in Humans
Source: PLoS Genet. 2015 Jan 24;11(1):e1004942. doi: 10.1371/journal.pgen.1004942 (PMC4305293; doi:10.1371/journal.pgen.1004942)

**A**

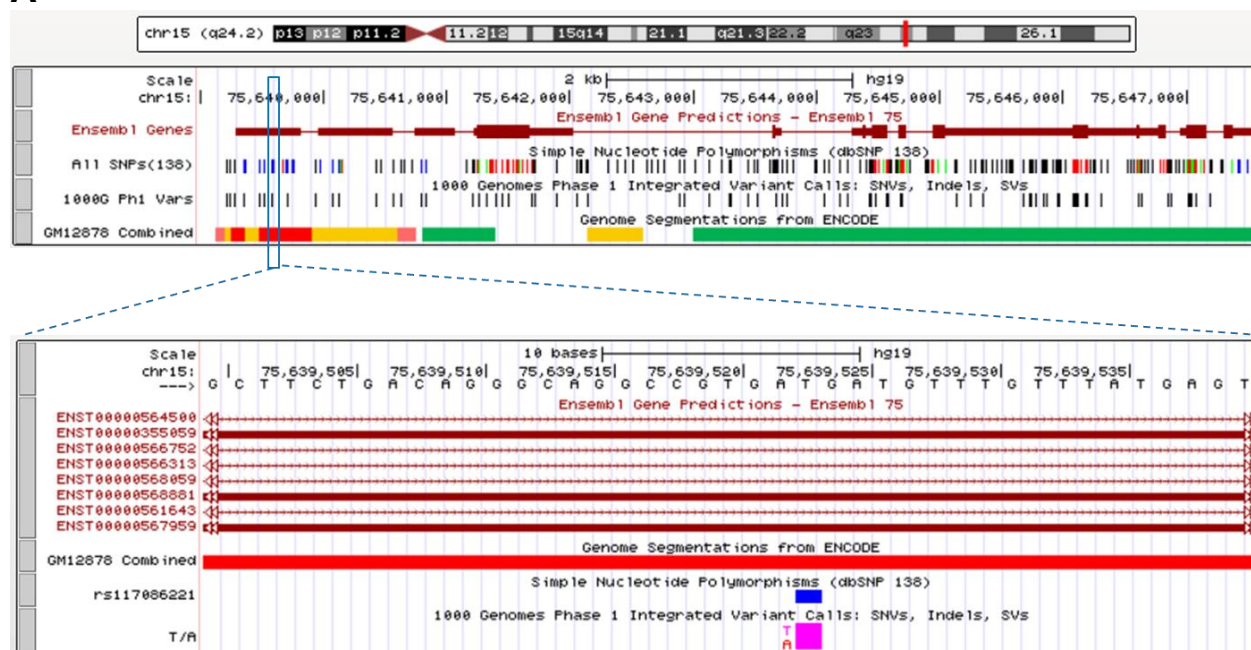

**B**

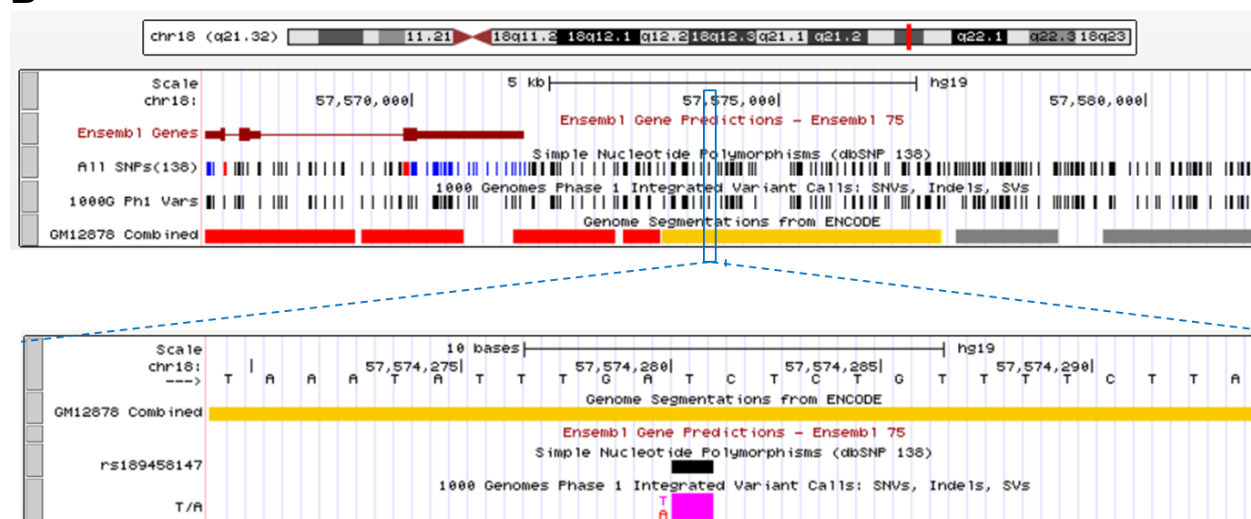

Supplement: S2 Fig — (A) Rs117086221 is located in the TSS region of gene NEIL1 in the individual NA12154. (B) Rs189458147 locates in the potential E region of gene PMAIP1 in the individual HG00122. (PDF) [file pgen.1004942.s002.pdf]

**A**

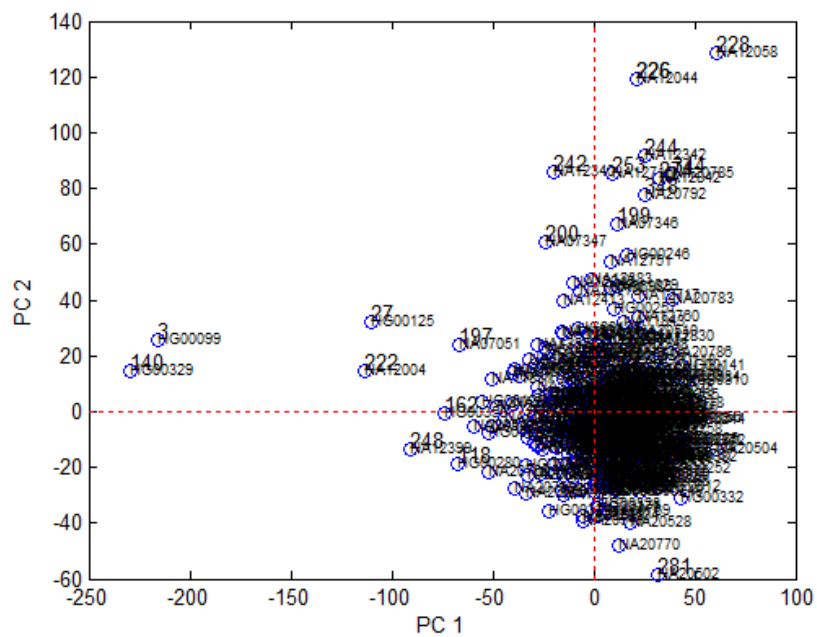

# B

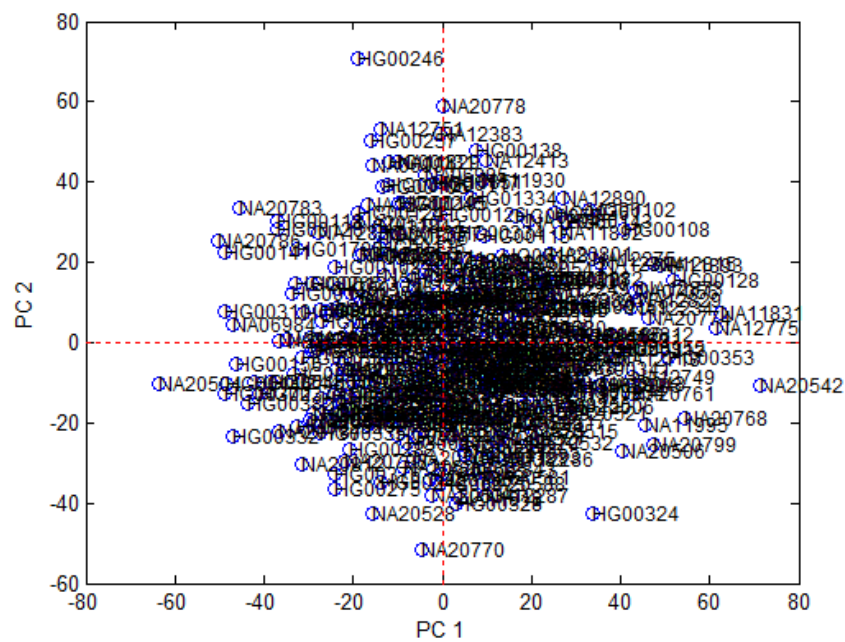

Supplement: S3 Fig — (A) A total of 19 outliers removed. They are: HG00099, HG00329, HG00125, NA12004, NA07051, HG00358, NA12399, HG00280, NA20502, NA07346, NA20792, NA12340, NA12716, NA12342, NA12842, NA20785, NA12044, NA12058, and NA07347 from populations of GBR, FIN, GBR, CEU, CEU, FIN, CEU, FIN, TSI, CEU, TSI, CEU, CEU, CEU, CEU, TSI, CEU, CEU, and CEU, respectively. (B) PCA result after the outliers are removed. (PDF) [file pgen.1004942.s003.pdf]
