# Supplementary material for: Aberrant Gene Expression in Humans
Source: PLoS Genet. 2015 Jan 24;11(1):e1004942. doi: 10.1371/journal.pgen.1004942 (PMC4305293; doi:10.1371/journal.pgen.1004942)
Supplement: S1 Table — (PDF) [file pgen.1004942.s004.pdf]

Table S1. GWAS gene sets that tend to be aberrantly expressed in LCLs of European descent.

GWAS catalog gene set	Number of genes[#]
**Genes tend to be aberrantly expressed**	
Adverse response to chemotherapy (neutropenia/leucopenia) (all platinum-based drugs)	2 / 6
Conduct disorder (symptom count)	4 / 7
Fasting insulin-related traits (interaction with BMI)	15 / 21
Metabolite levels (Dihydroxy docosatrienoic acid)	7 / 20
Obesity (extreme)	10 / 18
Retinal vascular caliber	14 / 18
Temperament	9 / 17
Thyroid hormone levels	14 / 28
**Genes tend not to be aberrantly expressed**	
Alcohol dependence	11 / 31
Metabolic syndrome	25 / 41

[#]Number of genes included in SSMD estimation / Number of genes in the GWAS catalog gene set.
