# Supplementary material for: Aberrant Gene Expression in Humans
Source: PLoS Genet. 2015 Jan 24;11(1):e1004942. doi: 10.1371/journal.pgen.1004942 (PMC4305293; doi:10.1371/journal.pgen.1004942)
Supplement: S2 Table — (PDF) [file pgen.1004942.s005.pdf]

**Table S2.** Gene sets with significant *diffSSMD* = $SSMD_{EUR}$-$SSMD_{YRI}$.

| | MSigDB gene set | # of genes | # of genes used in SSMD | Observed SSMD |
|---|---|---|---|---|
| **$diffSSMD_{obs}$ > $diffSSMD_{rand}$** | | | | |
| | PHOTORECEPTOR_CELL_MAINTENANCE | 10 | 8 | 13282.27 |
| | NIELSEN_MALIGNAT_FIBROUS_HISTIOCYTOMA_UP | 18 | 12 | 8588.03 |
| | WALLACE_PROSTATE_CANCER_DN | 6 | 4 | 5203.44 |
| | BERGER_MBD2_TARGETS | 7 | 2 | 1003.57 |
| **$diffSSMD_{obs}$ < $diffSSMD_{rand}$** | | | | |
| | GOLGI_MEMBRANE | 45 | 43 | 12167.03 |
| | VESICLE_COAT | 16 | 15 | 4060.43 |
| | INTEGRAL_TO_GOLGI_MEMBRANE | 10 | 10 | 2876.98 |
| | KINESIN_COMPLEX | 14 | 13 | 3482.33 |
| | GOLGI_APPARATUS_PART | 100 | 94 | 26243.37 |
| | ENDOCYTIC_VESICLE | 14 | 11 | 3066.16 |
| | COATED_VESICLE_MEMBRANE | 17 | 15 | 4060.43 |
| | TRANSPORT_VESICLE | 33 | 28 | 7491.02 |
| | INTEGRAL_TO_ORGANELLE_MEMBRANE | 50 | 48 | 14317.3 |
| | MEMBRANE_COAT | 17 | 16 | 4325.21 |
| | TRANS_GOLGI_NETWORK | 21 | 19 | 4744.92 |
| | GOLGI_ASSOCIATED_VESICLE | 29 | 26 | 7170.73 |
| | INTRINSIC_TO_GOLGI_MEMBRANE | 15 | 15 | 4531.93 |
| | COATED_MEMBRANE | 17 | 16 | 4325.21 |
| | TRANS_GOLGI_NETWORK_TRANSPORT_VESICLE | 13 | 12 | 2874.72 |
| | PHOSPHOLIPID_BIOSYNTHETIC_PROCESS | 39 | 38 | 9697.56 |
| | GLYCEROPHOSPHOLIPID_BIOSYNTHETIC_PROCESS | 30 | 29 | 7703.07 |
| | GLYCOLIPID_METABOLIC_PROCESS | 16 | 15 | 2599 |
| | VIRAL_GENOME_REPLICATION | 21 | 18 | 4830.73 |
| | PATTERN_SPECIFICATION_PROCESS | 31 | 20 | 4631.62 |
| | RESPONSE_TO_TOXIN | 10 | 7 | 270.64 |
| | VIRAL_INFECTIOUS_CYCLE | 32 | 26 | 7117.43 |
| | VIRAL_REPRODUCTIVE_PROCESS | 36 | 30 | 8402.15 |
| | POST_GOLGI_VESICLE_MEDIATED_TRANSPORT | 14 | 14 | 4122.54 |
| | MEMBRANE_LIPID_BIOSYNTHETIC_PROCESS | 49 | 47 | 10606.59 |
| | RAS_PROTEIN_SIGNAL_TRANSDUCTION | 66 | 61 | 9605.12 |
| | GLYCOSPHINGOLIPID_METABOLIC_PROCESS | 12 | 11 | 1131.99 |
| | NEGATIVE_REGULATION_OF_MAP_KINASE_ACTIVITY | 17 | 15 | 3907.94 |
| | GLYCEROPHOSPHOLIPID_METABOLIC_PROCESS | 46 | 44 | 10876.91 |
| | ESTABLISHMENT_AND_OR_MAINTENANCE_OF_CELL_POLARITY | 19 | 13 | 3864.83 |
| | INACTIVATION_OF_MAPK_ACTIVITY | 14 | 12 | 3095.47 |
| | SPHINGOLIPID_BIOSYNTHETIC_PROCESS | 10 | 9 | 926 |
| | REGULATION_OF_RAS_GTPASE_ACTIVITY | 11 | 11 | 3108 |
| | ACTIVE_TRANSMEMBRANE_TRANSPORTER_ACTIVITY | 122 | 91 | 28148.17 |
| | SUGAR_BINDING | 34 | 16 | 4145.68 |
| | SPECIFIC_RNA_POLYMERASE_II_TRANSCRIPTION_FACTOR_ACTIVITY | 35 | 31 | 7741.1 |
| | SULFOTRANSFERASE_ACTIVITY | 28 | 20 | 5935.63 |
| | OXIDOREDUCTASE_ACTIVITY_ACTING_ON_PEROXIDE_AS_ACCEPTOR | 12 | 12 | 3329.06 |
| | NEUROPEPTIDE_BINDING | 23 | 8 | 1173.58 |
| | NEUROPEPTIDE_RECEPTOR_ACTIVITY | 22 | 8 | 1173.58 |
| | NEUTRAL_AMINO_ACID_TRANSMEMBRANE_TRANSPORTER_ACTIVITY | 12 | 7 | 1776.72 |
| | PEPTIDE_BINDING | 85 | 51 | 16039.52 |
| | ACETYLGLUCOSAMINYLTRANSFERASE_ACTIVITY | 16 | 15 | 2975.02 |
| | NF_KAPPAB_BINDING | 11 | 11 | 2975.53 |
| | ACTIN_FILAMENT_BINDING | 25 | 25 | 6998.07 |
| | TRANSFERASE_ACTIVITY_TRANSFERRING_PENTOSYL_GROUPS | 20 | 17 | 5162.21 |
| | TRANSFERASE_ACTIVITY_TRANSFERRING_SULFUR_CONTAINING_GROUPS | 32 | 24 | 6177.74 |
| | CARBOXYLIC_ACID_TRANSMEMBRANE_TRANSPORTER_ACTIVITY | 44 | 27 | 7153.4 |
| | GALACTOSYLTRANSFERASE_ACTIVITY | 16 | 14 | 3551.61 |
| | AAGWWRNYGGC_UNKNOWN | 119 | 107 | 28387.78 |
| | AGCYRWTTC_UNKNOWN | 122 | 87 | 16729.68 |
| | ATGGYGGA_UNKNOWN | 102 | 92 | 28125.28 |
| | CCTNTMAGA_UNKNOWN | 127 | 102 | 32811.66 |
| | ACTACCT,MIR-196A,MIR-196B | 145 | 113 | 31626.25 |
| | KMCATNNWGGA_UNKNOWN | 90 | 77 | 21122.92 |

| | | | | |
|---|---|---|---|---|
| | RRCCGTTA_UNKNOWN | 87 | 74 | 23615.87 |
| | SNACANNNYSYAGA_UNKNOWN | 90 | 79 | 19106.18 |
| | TGTYNNNNNRGCARM_UNKNOWN | 86 | 68 | 23086.7 |
| | TNCATNTCCYR_UNKNOWN | 131 | 117 | 33594.9 |
| | MORF_TERF2IP | 112 | 110 | 32561.32 |
| | MORF_PAPSS1 | 101 | 100 | 29472.36 |
| | MORF_RUNX1 | 150 | 117 | 34955.63 |
| | MORF_SS18 | 61 | 59 | 17950.71 |
| | GCM_ERBB2IP | 64 | 61 | 19546.33 |
| | GCM_BMPR2 | 84 | 80 | 22228.1 |
| | GNF2_ATM | 30 | 28 | 8025.4 |
| | GNF2_CASP4 | 24 | 24 | 6469.52 |
| | GNF2_MSN | 24 | 24 | 6571.8 |
| | GNF2_PAK2 | 28 | 28 | 7839.01 |
| | GNF2_PTPN4 | 51 | 40 | 6740.23 |
| | GNF2_PTPRC | 68 | 67 | 20342.76 |
| | GNF2_RAP1B | 36 | 36 | 10316.35 |
| | GNF2_SNRK | 28 | 27 | 7832.82 |
| | GNF2_STAT6 | 79 | 79 | 23979.37 |
| | KEGG_GLYCOLYSIS_GLUCONEOGENESIS | 62 | 53 | 15518.48 |
| | KEGG_FATTY_ACID_METABOLISM | 42 | 33 | 8919.33 |
| | KEGG_GLYCEROPHOSPHOLIPID_METABOLISM | 77 | 64 | 19703.59 |
| | KEGG_GLYCOSPHINGOLIPID_BIOSYNTHESIS_LACTO_AND_NEOLACTO_SERIES | 26 | 21 | 5234.08 |
| | KEGG_GLYCOSPHINGOLIPID_BIOSYNTHESIS_GLOBO_SERIES | 14 | 12 | 1593.52 |
| | KEGG_GLYCOSPHINGOLIPID_BIOSYNTHESIS_GANGLIO_SERIES | 15 | 14 | 2324.53 |
| | KEGG_PROPANOATE_METABOLISM | 33 | 31 | 8209.5 |
| | KEGG_BIOSYNTHESIS_OF_UNSATURATED_FATTY_ACIDS | 22 | 22 | 5115.46 |
| | KEGG_PROSTATE_CANCER | 89 | 83 | 22999.38 |
| | BIOCARTA_BLYMPHOCYTE_PATHWAY | 11 | 11 | 2853.81 |
| | BIOCARTA_IL4_PATHWAY | 11 | 11 | 2993.47 |
| | BIOCARTA_MCALPAIN_PATHWAY | 25 | 23 | 7087.18 |
| | BIOCARTA_PPARA_PATHWAY | 58 | 48 | 5427.65 |
| | BIOCARTA_P38MAPK_PATHWAY | 40 | 39 | 11738.56 |
| | BIOCARTA_PML_PATHWAY | 17 | 16 | 4053.62 |
| | BIOCARTA_TEL_PATHWAY | 18 | 18 | 6510.63 |
| | BIOCARTA_ACTINY_PATHWAY | 20 | 19 | 5278.7 |
| | chr1p11 | 11 | 4 | 720.08 |
| | chr8p23 | 201 | 39 | 13126.04 |
| | chr9q32 | 45 | 26 | 6788.69 |
| | chrxq13 | 102 | 43 | 12290.93 |
| | chr9p22 | 61 | 18 | 3732.1 |
| | chr2q35 | 66 | 44 | 10933.41 |
| | MODULE_89 | 14 | 13 | 3134.48 |
| | MODULE_90 | 17 | 15 | 3334.59 |
| | MODULE_168 | 22 | 22 | 5893.79 |
| | MODULE_182 | 101 | 88 | 26595.14 |
| | MODULE_189 | 23 | 22 | 5163.49 |
| | MODULE_203 | 16 | 14 | 3340.15 |
| | MODULE_235 | 81 | 70 | 16624.16 |
| | MODULE_239 | 120 | 114 | 32611.37 |
| | MODULE_261 | 101 | 91 | 27362.39 |
| | MODULE_432 | 15 | 14 | 3771.95 |
| | MODULE_464 | 14 | 13 | 3639.27 |
| | MODULE_519 | 15 | 13 | 3244.43 |
| | MODULE_543 | 17 | 13 | 3082.26 |
| | MODULE_552 | 18 | 18 | 4693.43 |
| | PID_TELOMERASEPATHWAY | 68 | 67 | 19612.91 |
| | PID_HEDGEHOG_GLIPATHWAY | 48 | 46 | 12508.48 |
| | PID_TGFBRPATHWAY | 55 | 50 | 14630.74 |
| | REACTOME_GLYCOGEN_BREAKDOWN_GLYCOGENOLYSIS | 18 | 15 | 3997.26 |
| | REACTOME_PYRIMIDINE_CATABOLISM | 12 | 9 | 549.45 |
| | REACTOME_TETRAHYDROBIOPTERIN_BH4_SYNTHESIS_RECYCLING_SALVAGE_AND_REGULATION | 13 | 11 | 3080.34 |
| | REACTOME_PROTEOLYTIC_CLEAVAGE_OF_SNARE_COMPLEX_PROTEINS | 17 | 15 | 4213.4 |
| | REACTOME_ALPHA_LINOLENIC_ACID_ALA_METABOLISM | 12 | 11 | 1871.36 |

| | | | | |
|---|---|---|---|---|
| | REACTOME_ELEVATION_OF_CYTOSOLIC_CA2_LEVELS | 10 | 5 | 1053.85 |
| | REACTOME_METABOLISM_OF_NUCLEOTIDES | 72 | 66 | 18322.99 |
| | REACTOME_SPHINGOLIPID_METABOLISM | 69 | 59 | 18781.85 |
| | REACTOME_RECYCLING_PATHWAY_OF_L1 | 27 | 26 | 7619.25 |
| | REACTOME_AMINO_ACID_SYNTHESIS_AND_INTERCONVERSION_TRANSAMINATION | 17 | 16 | 4254.72 |
| | REACTOME_TRANSPORT_TO_THE_GOLGI_AND_SUBSEQUENT_MODIFICATION | 33 | 29 | 7564.11 |
| | REACTOME_N_GLYCAN_ANTENNAE_ELONGATION | 14 | 10 | 2384.54 |
| | REACTOME_KINESINS | 24 | 22 | 5924.76 |
| | REACTOME_N_GLYCAN_ANTENNAE_ELONGATION_IN_THE_MEDIAL_TRANS_GOLGI | 18 | 14 | 3433.84 |
| | REACTOME_CHOLESTEROL_BIOSYNTHESIS | 24 | 21 | 5678.3 |
| | REACTOME_PYRIMIDINE_METABOLISM | 24 | 21 | 3657.69 |
| | ZHONG_RESPONSE_TO_AZACITIDINE_AND_TSA_DN | 70 | 65 | 19101 |
| | LIU_VMYB_TARGETS_UP | 127 | 113 | 32555.06 |
| | NEWMAN_ERCC6_TARGETS_UP | 26 | 21 | 6248.71 |
| | RODRIGUES_DCC_TARGETS_DN | 121 | 112 | 35789.78 |
| | VECCHI_GASTRIC_CANCER_ADVANCED_VS_EARLY_DN | 138 | 93 | 16508 |
| | TIEN_INTESTINE_PROBIOTICS_2HR_DN | 88 | 85 | 22303.09 |
| | KIM_WT1_TARGETS_8HR_DN | 129 | 103 | 31243.64 |
| | LAU_APOPTOSIS_CDKN2A_DN | 5 | 4 | 973.36 |
| | CASTELLANO_HRAS_TARGETS_DN | 6 | 6 | 957.56 |
| | CASTELLANO_HRAS_AND_NRAS_TARGETS_DN | 8 | 7 | 1114.4 |
| | WANG_BARRETTS_ESOPHAGUS_UP | 51 | 37 | 10187.32 |
| | LUCAS_HNF4A_TARGETS_UP | 58 | 49 | 9702.12 |
| | KLEIN_TARGETS_OF_BCR_ABL1_FUSION | 45 | 40 | 11652.34 |
| | IWANAGA_E2F1_TARGETS_NOT_INDUCED_BY_SERUM | 6 | 6 | 1392.35 |
| | MYLLYKANGAS_AMPLIFICATION_HOT_SPOT_29 | 11 | 4 | 1207.76 |
| | MYLLYKANGAS_AMPLIFICATION_HOT_SPOT_18 | 8 | 5 | 1279.71 |
| | BOWIE_RESPONSE_TO_TAMOXIFEN | 18 | 18 | -1176.29 |
| | DAUER_STAT3_TARGETS_DN | 50 | 48 | 10800.47 |
| | KORKOLA_TERATOMA | 39 | 27 | 7927.57 |
| | TERAMOTO_OPN_TARGETS_CLUSTER_3 | 5 | 4 | 1049.74 |
| | TERAMOTO_OPN_TARGETS_CLUSTER_5 | 7 | 7 | 1684.51 |
| | HEIDENBLAD_AMPLICON_12P11_12_DN | 30 | 20 | 4203.83 |
| | MURATA_VIRULENCE_OF_H_PILORI | 24 | 20 | 5763.69 |
| | MAINA_VHL_TARGETS_DN | 18 | 14 | 2702.83 |
| | THEODOROU_MAMMARY_TUMORIGENESIS | 31 | 15 | 3597.49 |
| | KAUFFMANN_MELANOMA_RELAPSE_UP | 61 | 59 | 16517.77 |
| | GROSS_ELK3_TARGETS_DN | 32 | 25 | 7547.74 |
| | SCHAEFFER_PROSTATE_DEVELOPMENT_AND_CANCER_BOX6_DN | 5 | 2 | 443.28 |
| | COURTOIS_SENESCENCE_TRIGGERS | 6 | 6 | 1479.96 |
| | RICKMAN_HEAD_AND_NECK_CANCER_D | 37 | 21 | 7500.37 |
| | MORI_LARGE_PRE_BII_LYMPHOCYTE_DN | 58 | 56 | 16464.52 |
| | MORI_MATURE_B_LYMPHOCYTE_UP | 90 | 86 | 26604.64 |
| | NIKOLSKY_BREAST_CANCER_1Q32_AMPLICON | 13 | 12 | 3293.55 |
| | BYSTRYKH_SCP2_QTL | 6 | 4 | 619.58 |
| | KLEIN_PRIMARY_EFFUSION_LYMPHOMA_DN | 58 | 55 | 15315.08 |
| | PARK_HSC_VS_MULTIPOTENT_PROGENITORS_UP | 19 | 18 | 4654.95 |
| | SHIPP_DLBCL_VS_FOLLICULAR_LYMPHOMA_DN | 45 | 40 | 9414.39 |
| | REN_BOUND_BY_E2F | 61 | 61 | 18365.62 |
| | GALINDO_IMMUNE_RESPONSE_TO_ENTEROTOXIN | 85 | 81 | 26394.45 |
| | FAELT_B_CLL_WITH_VH_REARRANGEMENTS_UP | 48 | 48 | 12734.2 |
| | NEMETH_INFLAMMATORY_RESPONSE_LPS_UP | 88 | 82 | 24735.13 |
| | XU_RESPONSE_TO_TRETINOIN_UP | 16 | 14 | 3396.55 |
| | GREENBAUM_E2A_TARGETS_UP | 33 | 32 | 8452.65 |
| | ZHANG_RESPONSE_TO_CANTHARIDIN_UP | 19 | 17 | 4657.55 |
| | BROWNE_INTERFERON_RESPONSIVE_GENES | 68 | 64 | 13894.72 |
| | DASU_IL6_SIGNALING_SCAR_UP | 30 | 27 | 8056.83 |
| | CHIBA_RESPONSE_TO_TSA | 50 | 42 | 12412.14 |
| | EHRLICH_ICF_SYNDROM_DN | 15 | 14 | 3931.79 |
| | WEIGEL_OXIDATIVE_STRESS_BY_HNE_AND_TBH | 60 | 53 | 16019.48 |
| | ZHU_CMV_24_HR_UP | 93 | 90 | 23697.2 |
| | NIELSEN_LEIOMYOSARCOMA_UP | 18 | 14 | -602.41 |
| | GENTILE_UV_RESPONSE_CLUSTER_D8 | 40 | 38 | 10431.4 |

| | | | | |
|---|---|---|---|---|
| | GENTILE_RESPONSE_CLUSTER_D3 | 61 | 59 | 17748.07 |
| | TSENG_ADIPOGENIC_POTENTIAL_UP | 30 | 22 | 6353.8 |
| | DURCHDEWALD_SKIN_CARCINOGENESIS_UP | 88 | 68 | 20112.66 |
| | GAVIN_FOXP3_TARGETS_CLUSTER_T4 | 94 | 88 | 27334.62 |
| | GAVIN_IL2_RESPONSIVE_FOXP3_TARGETS_DN | 5 | 4 | 749.06 |
| | MCGOWAN_RSP6_TARGETS_DN | 5 | 5 | 1094.58 |
| | MOSERLE_IFNA_RESPONSE | 31 | 31 | 9180.06 |
| | BERNARD_PPAPDC1B_TARGETS_UP | 40 | 35 | 10925.22 |
| | CHUNG_BLISTER_CYTOTOXICITY_DN | 44 | 41 | 11565.73 |
| | SHARMA_PILOCYTIC_ASTROCYTOMA_LOCATION_DN | 7 | 4 | 1182.49 |
| | HUANG_DASATINIB_RESISTANCE_DN | 69 | 59 | 12873.63 |
| | CHAUHAN_RESPONSE_TO_METHOXYESTRADIOL_DN | 102 | 98 | 29003.88 |
| | MARTINELLI_IMMATURE_NEUTROPHIL_UP | 11 | 3 | 372.46 |
| | HOFFMANN_SMALL_PRE_BII_TO_IMMATURE_B_LYMPHOCYTE_UP | 70 | 63 | 19582.94 |
| | LEE_EARLY_T_LYMPHOCYTE_DN | 57 | 51 | 15821.05 |
| | SHEDDEN_LUNG_CANCER_GOOD_SURVIVAL_A5 | 70 | 58 | 16674.65 |
| | FERRANDO_HOX11_NEIGHBORS | 23 | 18 | 5263.15 |
| | ZHAN_EARLY_DIFFERENTIATION_GENES_DN | 42 | 40 | 11082.27 |
| | MILI_PSEUDOPODIA | 43 | 43 | 12697.83 |
| | IRITANI_MAD1_TARGETS_UP | 13 | 9 | 1018.97 |
| | ZHAN_LATE_DIFFERENTIATION_GENES_DN | 16 | 15 | 3173.69 |
| | BOYAULT_LIVER_CANCER_SUBCLASS_G1_DN | 40 | 37 | 11819.47 |
| | CHIANG_LIVER_CANCER_SUBCLASS_UNANNOTATED_UP | 85 | 68 | 20471.15 |
| | MIKKELSEN_IPS_ICP_WITH_H3K27ME3 | 54 | 27 | 9328.12 |
| | MARSON_FOXP3_TARGETS_DN | 54 | 49 | 14468.09 |
| | WINNEPENNINCKX_MELANOMA_METASTASIS_DN | 46 | 26 | -728.51 |
| | TSAI_DNAJB4_TARGETS_UP | 13 | 12 | 3266.59 |
| | ZHAN_MULTIPLE_MYELOMA_CD1_DN | 45 | 42 | 12073.8 |
| | KYNG_RESPONSE_TO_H2O2_VIA_ERCC6_DN | 46 | 43 | 10219.1 |
| | DAZARD_UV_RESPONSE_CLUSTER_G24 | 28 | 19 | -944.96 |
| | RUAN_RESPONSE_TO_TROGLITAZONE_DN | 19 | 14 | 3535.99 |
| | KIM_ALL_DISORDERS_DURATION_CORR_UP | 9 | 7 | 1825.02 |
| | JOHNSTONE_PARVB_TARGETS_1_DN | 63 | 50 | 6177.03 |
| | DELPUECH_FOXO3_TARGETS_UP | 68 | 62 | 18823.22 |
| | KASLER_HDAC7_TARGETS_2_DN | 32 | 30 | 8681.28 |
| | JUBAN_TARGETS_OF_SPI1_AND_FLI1_UP | 115 | 107 | 32090.64 |
| | KOHOUTEK_CCNT2_TARGETS | 58 | 39 | 11809.4 |
| | LE_NEURONAL_DIFFERENTIATION_DN | 19 | 17 | 4403.91 |
| | HOLLEMAN_ASPARAGINASE_RESISTANCE_ALL_DN | 25 | 23 | 6467.86 |
| | SMIRNOV_RESPONSE_TO_IR_2HR_DN | 55 | 54 | 15749.72 |
| | BCAT_BILD_ET_AL_DN | 46 | 44 | 13511.4 |
| | EIF4E_UP | 100 | 79 | 24878.26 |
| | PDGF_ERK_DN.V1_DN | 149 | 129 | 38457.45 |
| | HORTON_SREBF_TARGETS | 25 | 23 | 6436.94 |
| | GSE15659_NAIVE_CD4_TCELL_VS_RESTING_TREG_UP | 200 | 146 | 27222.29 |
| | GSE15659_NAIVE_CD4_TCELL_VS_ACTIVATED_TREG_UP | 200 | 133 | 36038.03 |
| | GSE15659_RESTING_VS_ACTIVATED_TREG_UP | 200 | 120 | 22963.11 |
| | GSE17974_IL4_AND_ANTI_IL12_VS_UNTREATED_1H_ACT_CD4_TCELL_UP | 200 | 113 | 34236.76 |
| | GSE34205_HEALTHY_VS_RSV_INF_INFANT_PBMC_DN | 200 | 148 | 42489.3 |
