# Supplementary material for: Aberrant Gene Expression in Humans
Source: PLoS Genet. 2015 Jan 24;11(1):e1004942. doi: 10.1371/journal.pgen.1004942 (PMC4305293; doi:10.1371/journal.pgen.1004942)
Supplement: S3 Table — (PDF) [file pgen.1004942.s006.pdf]

Table S3. *P*-values of Kolmogorov-Smirnov test for the normalized relative mean difference (RMD) between "L-SSMD" and "S-SSMD" genes in monozygotic (MZ) and dizygotic (DZ) twins.

	DZ S-SSMD	MZ outlier	DZ L-SSMD
MZ S-SSMD	5.42E-06	2.47E-15	2.21E-64
DZ S-SSMD		3.60E-04	3.00E-34
MZ L-SSMD			5.59E-23
